# A Novel MBAS-RF Approach to Predict Mechanical Properties of Geopolymer-Based Compositions

**DOI:** 10.3390/gels9060434

**Published:** 2023-05-24

**Authors:** Shuzhao Chen, Mengmeng Zhou, Xuyang Shi, Jiandong Huang

**Affiliations:** 1 School of Mines, China University of Mining and Technology, Xuzhou 221116, China; 2 School of Civil Engineering, Guangzhou University, Guangzhou 510006, China

**Keywords:** gels, concrete, hybrid machine learning model, compressive strength

## Abstract

Using gels to replace a certain amount of cement in concrete is conducive to the green concrete industry, while testing the compressive strength (CS) of geopolymer concrete requires a substantial amount of substantial effort and expense. To solve the above issue, a hybrid machine learning model of a modified beetle antennae search (MBAS) algorithm and random forest (RF) algorithm was developed in this study to model the CS of geopolymer concrete, in which MBAS was employed to adjust the hyperparameters of the RF model. The performance of the MBAS was verified by the relationship between 10-fold cross-validation (10-fold CV) and root mean square error (RMSE) value, and the prediction performance of the MBAS and RF hybrid machine learning model was verified by evaluating the correlation coefficient (R) and RMSE values and comparing with other models. The results show that the MBAS can effectively tune the performance of the RF model; the hybrid machine learning model had high R values (training set R = 0.9162 and test set R = 0.9071) and low RMSE values (training set RMSE = 7.111 and test set RMSE = 7.4345) at the same time, which indicated that the prediction accuracy was high; NaOH molarity was confirmed as the most important parameter regarding the CS of geopolymer concrete, with the importance score of 3.7848, and grade 4/10 mm was confirmed as the least important parameter, with the importance score of 0.5667.

## 1. Introduction

**Figure 1:**
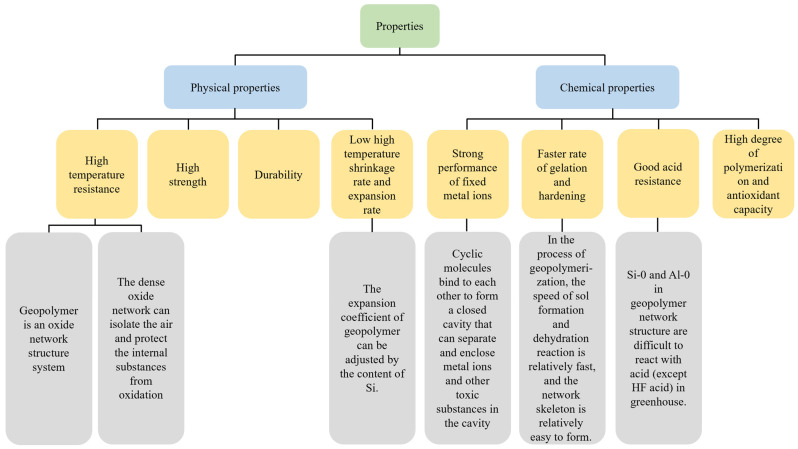
Properties of gels.

Gels are the elastic semi-solid materials with elasticity formed by the increase in viscosity of polymer solution or sol under certain conditions, which can use natural minerals containing silicon aluminate substances (such as metakaolin), industrial solid waste (such as fly ash, slag, steel slag, and various tailings, etc.) as the main material, and can be prepared at a normal temperature or slightly high temperature by the action of an alkali activator. Compared to the preparation of geological polymers, cement is made of clay and limestone mixed at high temperatures, and as the main cementing material of concrete, it has strong irreplaceability [1,2,3,4]. The cement production process, due to the use of limestone as the main raw material, will emit a large amount of CO_2_ into the air, as well as harmful gases and dust, such as SO_2_ and NO_X_, which aggravate the greenhouse effect and cause environmental pollution. [5,6,7]. In addition, the preparation of cement needs the support of electric power resources, which mainly take coal as the basic fuel, and the combustion of coal is accompanied by the production of industrial waste, such as fly ash and slag, which will not only affect the ecological environment, but also occupy a large number of land resources [8,9,10,11]. The above analysis proves that the mass use of cement is not conducive to the sustainable development of the construction industry. Hence, it is urgent for sustainable development of the concrete industry to find or develop a kind of environment-friendly cementitious material that can be used to partially or completely replace cement in concrete [12,13]. In response to the above problems, engineers creatively proposed to use industrial waste slag and other materials with certain activities as cementing materials to partially or completely replace the cement in concrete, so as to alleviate the environmental pollution and resource consumption caused by cement production and promote the sustainable development of the construction industry [14,15,16,17,18]. Gel is an inorganic aluminosilicate compound formed from alkali-activated industrial waste, metakaolin, and other materials containing a large amount of Si and Al, which does not exist in the high-temperature calcination link in the production process, so it will not release harmful components into the air, nor will it produce solid waste that pollutes the environment due to coal combustion [19,20,21,22,23]. Table 1 and Figure 1 show the classification and properties of gels, respectively.

The application of gels in concrete became a hot topic, and many researchers focus on the study of compressive strength, which is an important property of geopolymer concrete [24]. In order to explore the impact of high-temperature exposure on the strength and quality loss of ordinary concrete and geopolymer concrete, Indu et al. [25] carried out research using XRD, X-ray, and SEM methods, and the results show that the strength loss of ordinary concrete under high temperature exposure was about 18% higher than that of geopolymer concrete. Khoa et al. [26] proposed to evaluate the strength of fly ash-based geopolymer concrete using the deep neural network (DNN) and res net architecture to solve the shortcomings of the long time and high cost of traditional laboratory experiment methods. Yaswanth et al. [27] developed a neural network method for predicting the CS of geopolymer concrete and verified the accuracy of the developed model by comparing the consistency between the predicted and actual values. Awoyera et al. [28] used gene expression programming (GEP) and an artificial neural network (ANN) to predict the CS, splitting, and flexural strength of gels self-compacting concrete with mineral admixtures, and the results show that GEP and ANN had high prediction effects. Quang et al. [29] evaluated the influence of sodium hydroxide/sodium ratio, fly ash/aggregate ratio, the effect of alkali activator/fly ash ratio, concentration of sodium hydroxide, curing time, and temperature on the CS of geopolymer concrete, using ANN, deep neural networks (DNN), and deep residual network (ResNet) methods, and the results show that the fly ash/aggregate ratio had the greatest effect on the CS of geopolymer concrete. Ghosh et al. [30] compared the prediction effects of linear regression (LR), decision tree (DT), RF, and support vector machine (SVM) on the CS of geopolymer concrete, emphasized the replacement level of fly ash, curing time and curing environment on the CS, and proved the feasibility of machine learning models in evaluating the CS.

**Figure 2:**
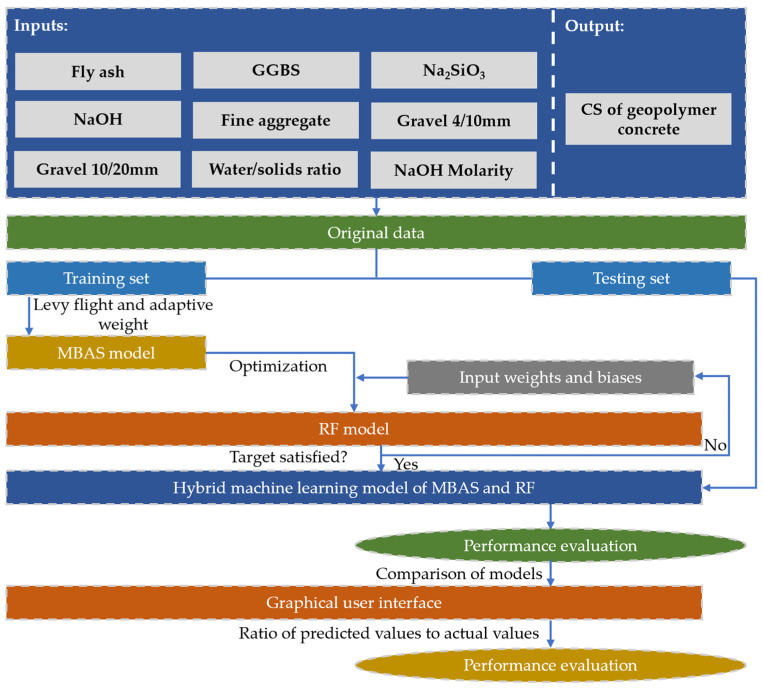
Research plan opted for this study.

Although some researchers realized the superiority of machine learning models in evaluating the CS of geopolymer concrete, most of them use single machine learning models, and the prediction accuracy of single machine learning models is lower than that of hybrid machine learning models. In order to solve the above problems, the adaptive inertia weight and the Levy flight method were employed to develop a modified beetle antennae search (MBAS) algorithm, and then it was combined with the random forest (RF) model to establish a MBAS-RF model, in which MBAS was employed to adjust the hyperparameters of the RF model, and RF was used to predict the CS of geopolymer concrete. Finally, the effectiveness of RF-MBAS was verified by comparing the prediction results of RF-MBAS with other hybrid machine learning models, and a graphical user interface (GUI) for evaluating the CS of geopolymer concrete was designed based on RF-MBAS. The research plan was selected for this study, as shown in Figure 2.

## 2. Methodology

### 2.1. Data Collection and Analysis

**Figure 3:**
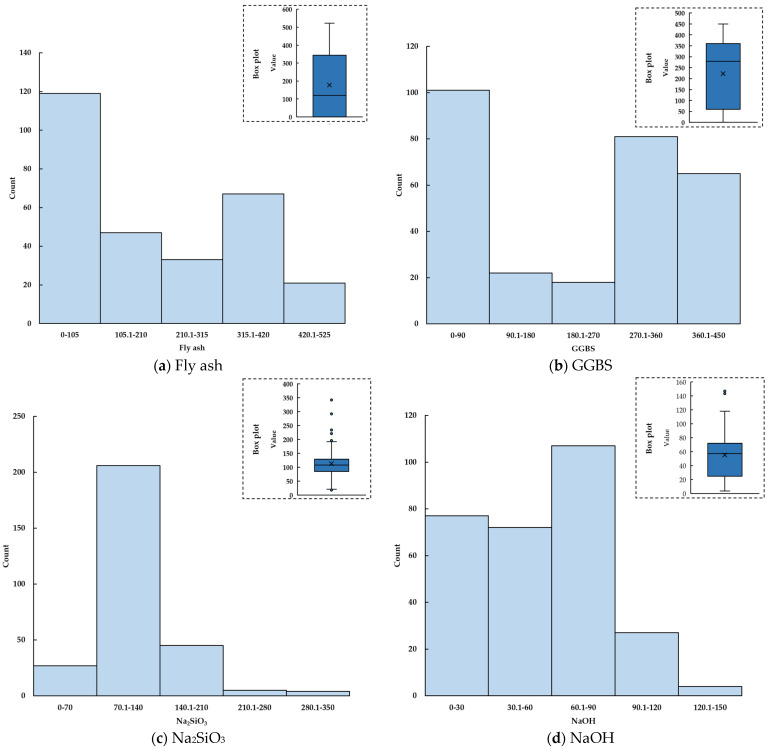

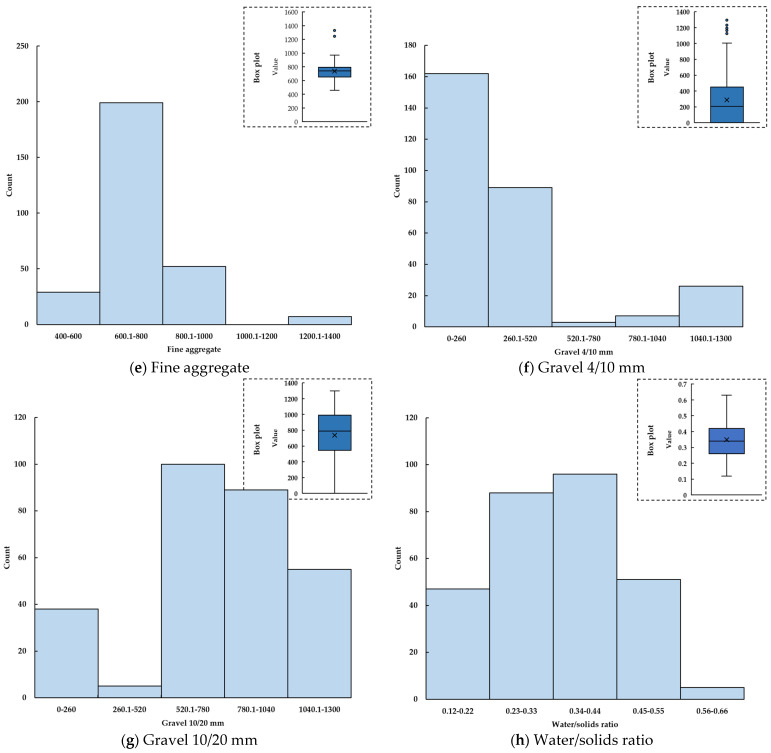

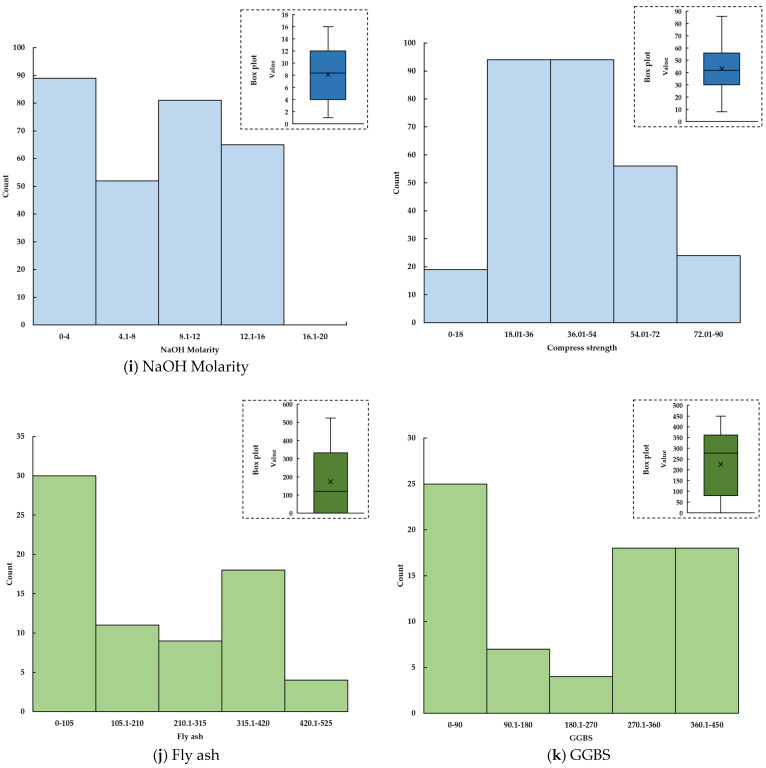

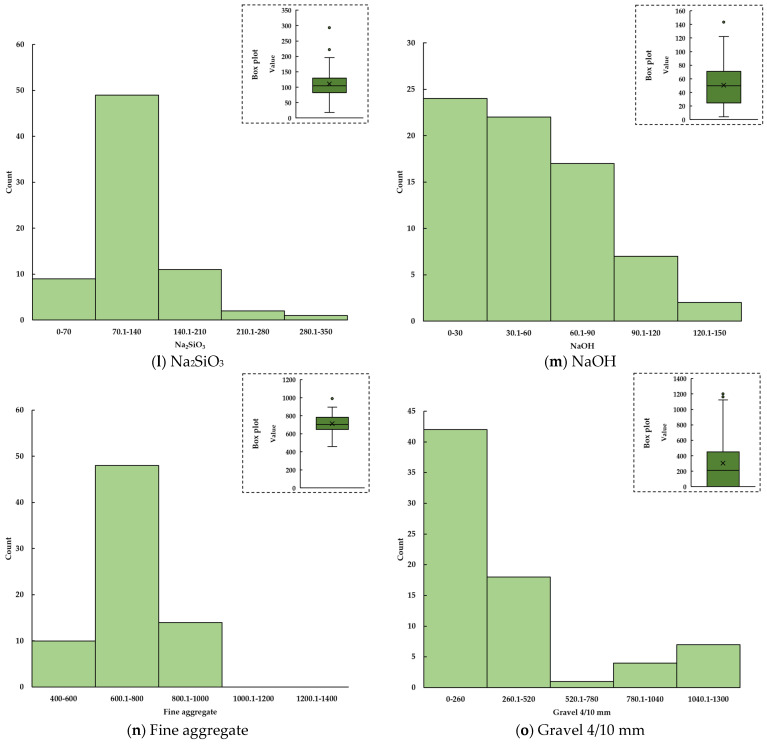

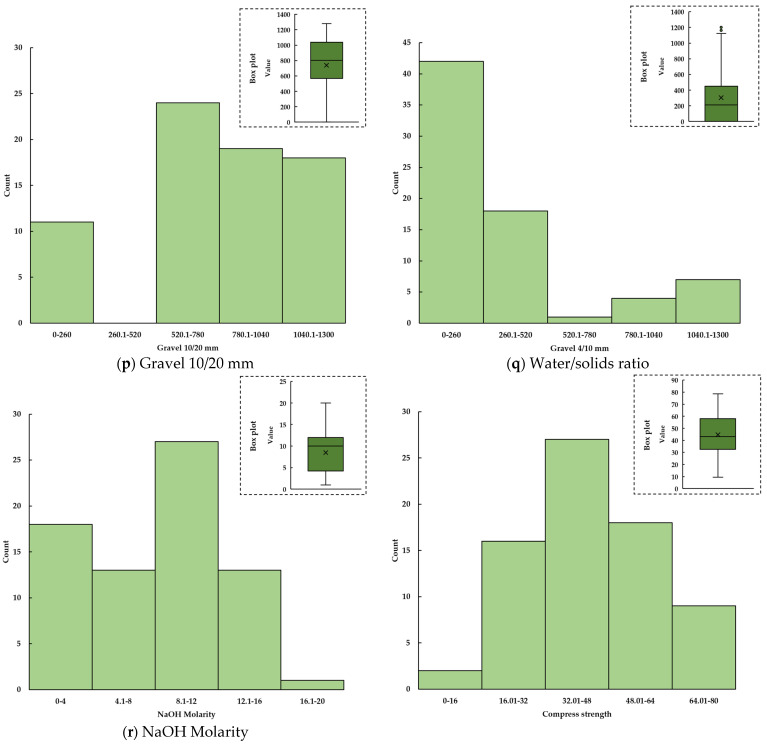
Frequency distribution histograms of input variables (training set: (**a**–**i**), test: set: (**j**–**r**)).

The raw materials of gels include a variety of alkali excitation materials and industrial solid waste, and the content of these raw materials affects the performance of the gels. Sufficient Na^+^ and OH^−^ are required to complete the whole process of the polymerization of gels, and the content of Na^+^ and OH^−^ has a certain impact on the strength of gels [31]. Considering the above two reasons and the influence of the characteristics and ratio of raw material on the CS of concrete, this study finally decided to use ground-granulated blast-furnace slag (GGBS), Na_2_SiO_3_, fly ash, gravel stones with sizes of 4–10 mm and 10–20 mm, water/solids ratio, NaOH, NaOH molarity, and fine aggregate as the input variables to evaluate the CS of geopolymer concrete in this study, and referred to the studies of Rafet et al. to form a database containing 359 datasets [32,33,34,35,36,37,38,39,40,41,42,43,44,45,46,47,48,49,50,51,52,53,54,55,56,57,58,59,60,61,62,63,64,65,66,67,68,69,70,71,72,73,74,75,76,77,78,79]. The datasets were randomly divided into two parts: training set (80%) and test set (20%), among which the training set was used for model training, and the test set was used for the evaluation of model effect [75,76]. The data distribution and statistical analysis of variables are shown in Figure 3 and Table 2.

**Figure 4:**
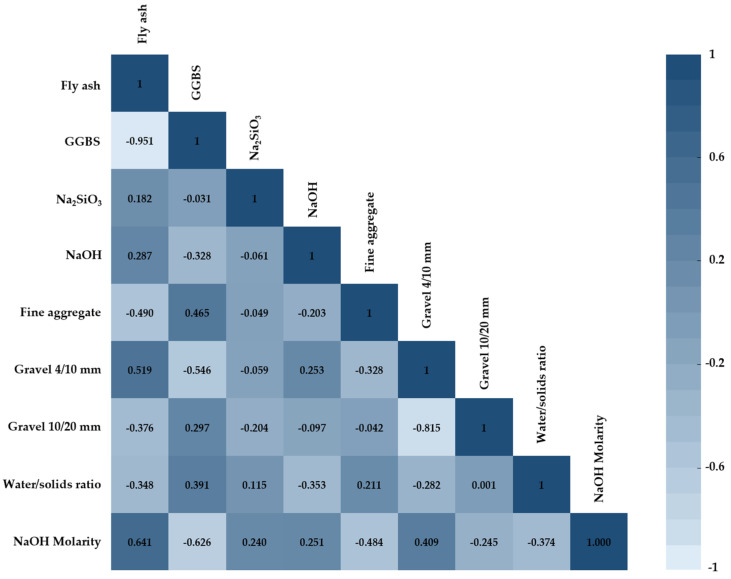
Pearson correlation coefficient between the input parameters.

Before the training of models, this study analyzed the Pearson correlation coefficient between input variables for evaluating the CS of geopolymer concrete with SPSS software, and the results are shown in Figure 4. It can be seen from the figure that there exists the highest negative correlation between fly ash and GGBS at −0.951, and the subsequent highest negative correlation is between GGBS and NaOH molarity, with r equal to −0.626. On the other hand, the high positive correlation belonged to fly ash and NaOH molarity. Interestingly, the correlation between the water/solids ratio and gravel 10/20 mm is the lowest, only 0.01, which is close to 0. On the whole, there is no high correlation between variables, so the evaluation of model effect will not be affected by the occurrence of multicollinearity among input variables.

### 2.2. Analysis of the Machine Learning Models Employed

#### 2.2.1. Beetle Antennae Search (BAS)

BAS is widely used because it can realize optimization without knowing specific function expressions and any gradient information [80,81,82]. The steps of BAS can be summarized as follow:

(1) Initialize the location of the beetle, and the formula is as follows:

(1) $$\partial =\frac{rnd(b,1)}{\left\|rnd(b,1)\right\|}$$

where rnd(⋅) represents the random function and b represents the dimension of the search space.

(2) Determine the distance between the whiskers of the beetle:

(2) $$d_{i}=\frac{\delta^{i}}{c}$$

where $d_{i}$ is the distance between the two whiskers of the ith beetle, $\delta^{i}$ is the step length of the ith beetle, and c is a constant.

(3) Simulate the search behavior of the left and right whiskers:

(3) $$x_{l}=x_{i}-d_{i}\alpha$$

(4) $$x_{r}=x_{i}+d_{i}\alpha$$

where $x_{l}$ and $x_{r}$, respectively, represent the left whisker and right whisker of beetles, and $x_{i}$ represents the position of the ith beetle.

(4) The behavior of the beetle can be determined by the following equation:

(5) $$x_{i+1}=x_{i}+\delta^{i}\alpha sign(f(x_{r})-f(x_{l}))$$

where f(⋅) is an adaptive function and sign(⋅) is a symbolic function. The updated formula of step size is:

(6) $$\delta^{i+1}=\eta \delta^{i}$$

where η represents the attenuation coefficient of the step.

Although BAS is an efficient intelligent search algorithm, as the step size of the beetle remains unchanged or decreases with the increase in the number of iterations, the algorithm has certain limitations. If the given step size is large, it may jump out of the local optimum value. If the given step size is too small, the algorithm may converge too slowly or fall into the local optimum. In order to solve the limitations of BAS, this study proposes to adjust the traditional BAS step size by using Levy flight and self-inertia weight to obtain MBAS, which can effectively adjust the adjustment step size and reduce oscillation.

(1) Levy flight

In order to solve the problem that the traditional BAS is prone to fall into local optimization due to the step size adjustment strategy, this study uses Levy flight to interfere with the population so that the algorithm can expand the search range in the early global exploration stage and prevent premature algorithm. Levy flight improves BAS using the following formula:

(7) $$\delta^{\left(i\right)}=\alpha \left|Levy\right|⊗\delta^{\left(i-1\right)}$$

in which $\delta^{i}$ represents the size of the step at the ith iteration, α represents the randomization parameter, and α∈[0,1], ⊗ represents the term multiplication, |Levy| represents the Levy distribution with infinite variance, and infinity is defined as $Levyu=t^{-\lambda },(1<\lambda \leq 3)$. Obtain the Levy flight as:

(8) $$\left|f^{\left(i\right)}-f^{\left(i-1\right)}\right|<\mu (f_{w}-f_{b})$$

in which μ is the coefficient and $\mu =10^{-5}$, $f_{w}$ and $f_{b}$ represent the worst and best historical adaptation value, respectively.

(2) Self-adaptive inertia weight

The adaptive weight in this study is represented by monotone decline equation, and the formula is as follows:

(9) $$\delta^{i+1}=\eta^{i}\times \delta^{i}$$

where $\delta^{i}$ represents the step size of the position, $\eta^{i}$ represents the adaptive weight value, and is defined as:

(10) $$\eta^{i}=(1-\alpha )0.95+\alpha \frac{f_{w}^{i}-f^{i}}{f_{w}^{i}-f_{b}^{i}}$$

where $f^{i}$ represents the fitting equation of the location, $f_{b}^{i}$ and $f_{w}^{i}$ represent the best and the worst fit value, respectively, ((1−α)0.95) represents the inertia weight, $\left(\alpha \frac{f_{w}^{i}-f^{i}}{f_{w}^{i}-f_{b}^{i}}\right)$ represents the adaptive feature, α represents the hyperparameter of the trade-off between the inertia weight and the adaptive feature, and α is the coefficient and is taken as 0.2 in this study.

#### 2.2.2. Random Forests (RF)

**Figure 5:**
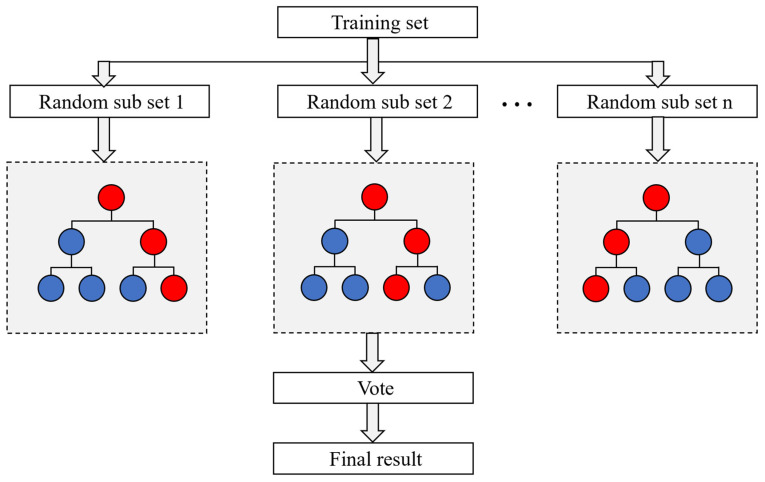
Schematic diagram of RF.

The idea of integrated learning is to solve the inherent defects of a single model or a group of parameter models, so as to integrate more models, learn the strengths of each model, and avoid boundedness [23]. RF is a commonly integrated learning algorithm, and the main idea of it is to obtain the final result by combining multiple weak classifiers and voting or averaging [83]. “Random” and “forest” make the model have high accuracy, strong generalization ability, and anti-overfitting [84]. Figure 5 shows the schematic diagram of RF.

### 2.3. Evaluation of Predictive Performance

**Figure 6:**
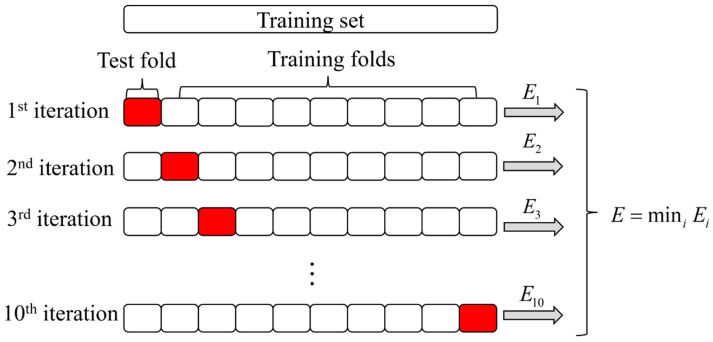
Structure diagram of 10-fold CV.

Before model training, this study used MBAS to adjust the hyperparameters of RF to improve the accuracy of model prediction [85]. To verify the effect of hyperparameter tuning and select the best hyperparameters, 10-fold CV can be used. The idea of 10-fold CV is to randomly divide the training set into ten parts, select one part as the verification set in turn, and the remaining nine parts as the training set. This process is repeated ten times, and the structure diagram of 10-fold CV is shown in Figure 6.

RMSE and R values were used to evaluate the error between the predicted and the actual values to verify the prediction accuracy of the models for the CS of geopolymer concrete, and the RMSE value can be determined by the following equation [86]:

(11) $$\mathrm{RMSE}=\sqrt{\frac{\sum_{i=1}^{n}{\left(y_{i}^{*}-y_{i}\right)}^{2}}{n}}$$

where *n* represents the number of datasets, $y_{i}^{*}$ and $y_{i}$ represent the predicted and actual CS of geopolymer concrete, respectively.

The formula for R can be summarized as follows [84,85,86,87,88,89]:

(12) $$R=\frac{\sum_{i=1}^{N}\left(y_{i}^{*}-\bar{y^{*}}\right)(y_{i}-\bar{y})}{\sqrt{\sum_{i=1}^{n}{\left(y_{i}^{*}-\bar{y^{*}}\right)}^{2}}\sqrt{\sum_{i=1}^{N}{\left(y_{i}-\bar{y}\right)}^{2}}}$$

where $\bar{y^{*}}$ and $\bar{y}$ represent the average of predicted and actual values, respectively.

## 3. Analysis of Results

### 3.1. Performance of MBAS

**Figure 7:**
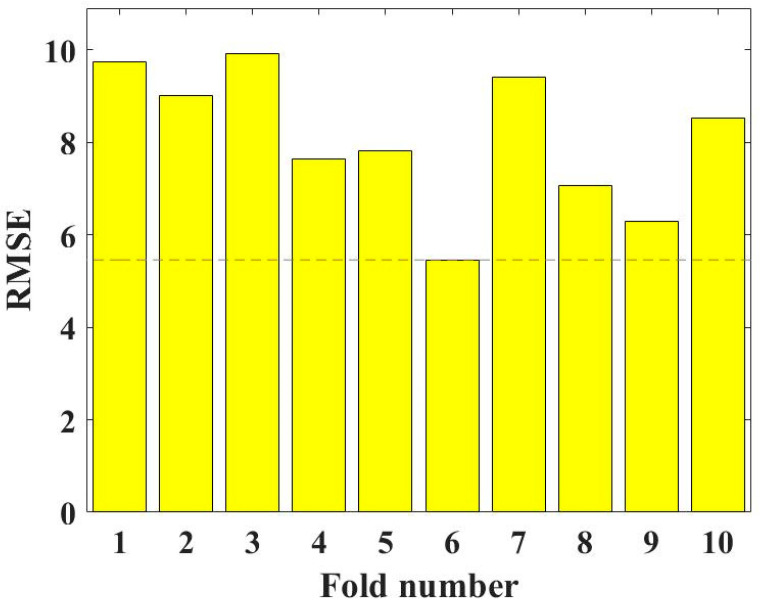
RMSE results of the hyperparameter tuning.

The 10-fold CV can effectively verify the hyperparameter tuning effect of models [82,89,90] so as to obtain the best hyperparameters. The hyperparameter tuning effect of MBAS on RF is shown in Figure 7. Obviously, the RMSE values are low at each fold, and the lowest RMSE values are reached at the sixth fold, demonstrating that the MBAS can effectively adjust the RF model used to evaluate the CS of geopolymer concrete.

**Figure 8:**
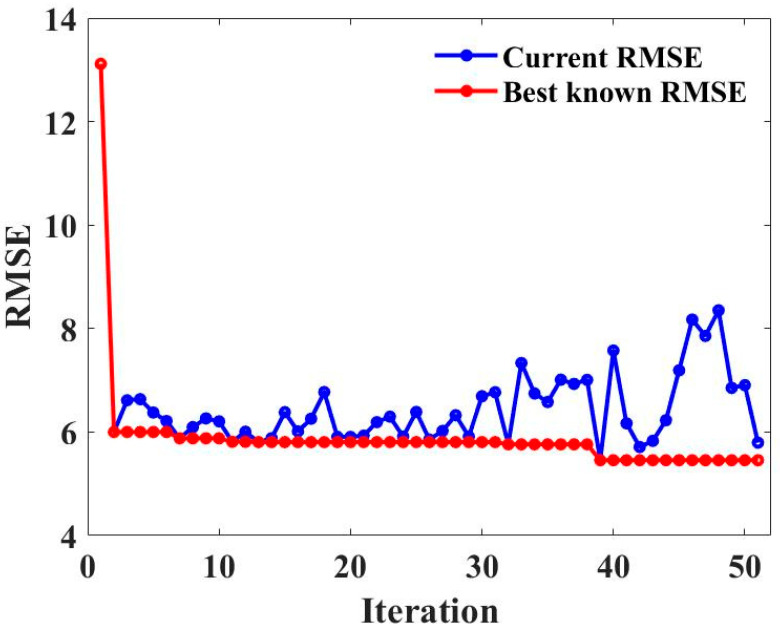
RMSE values regarding varying iteration numbers of RF.

Figure 8 shows the change in RMSE values for varying iteration numbers. With the increases in iterations, the RMSE values drop sharply and become stable before the number of iterations reaches 5. It is proven that the search efficiency of MBAS improved by Levy flight and adaptive weight is reliable [91,92].

### 3.2. Evaluating the Prediction Performance

**Figure 9:**
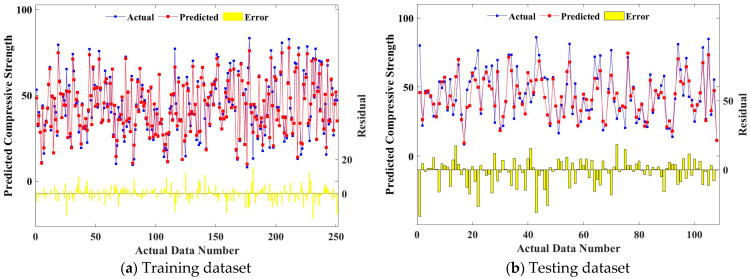
Relationship between predicted values and measured values of RF.

Figure 9 shows the prediction effect of RF-MBAS on the CS of geopolymer concrete, and the bar chart represents the error between the predicted and actual values. As can be seen from the figure, the consistency between the predicted and actual values of the training set and the test set is very high, and only some of the predicted values deviated from the actual values. On the whole, the prediction accuracy of RF-MBAS for the CS of geopolymer concrete is high.

**Figure 10:**
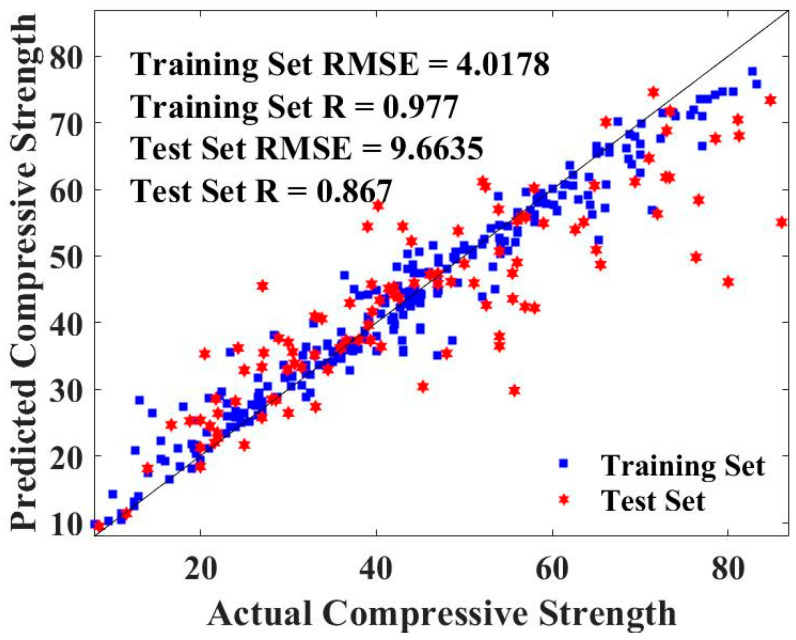
Fitting effect between predicted values and actual values of RF.

Figure 10 represents the fitting effect between the predicted values and the actual values, as well as the quantitative analysis result of the predicted effect. The fitting effect between the predicted values and the actual values is almost close to the perfect fitting curve with R = 1, and both the training set and test set have high R values (0.977 and 0.867) and low RMSE values (4.0178 and 9.6635), proving that RF-MBAS is a hybrid machine learning model that can accurately predict the CS of geopolymer concrete.

### 3.3. Models Comparison

**Figure 11:**
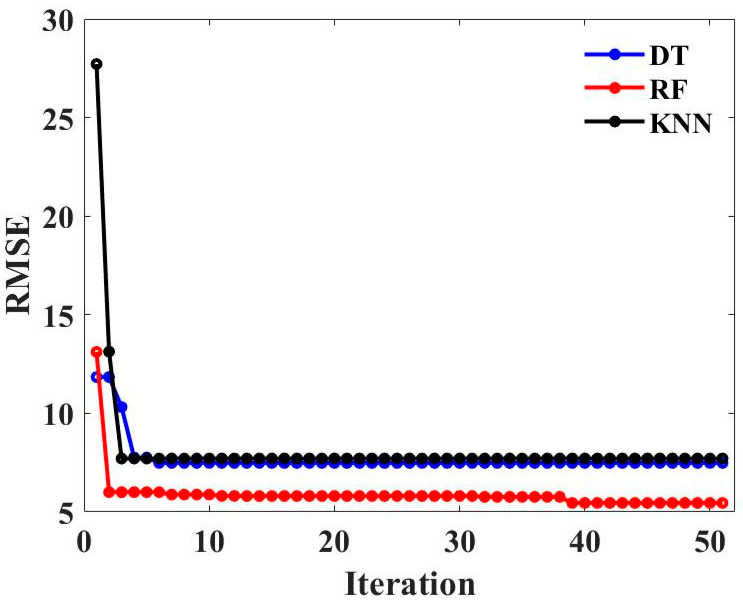
RMSE values regarding varying iteration numbers of DT, RF, and KNN.

Decision tree (DT) and K nearest neighbor (KNN) are often used to solve classification and prediction problems in practical applications due to their advantages, such as high efficiency, high precision, and others. Hence, this study chooses to compare the prediction accuracy of RF and DT and KNN optimized by MBAS for the CS of geopolymer concrete to verify that RF-MBAS exhibits the highest predictive accuracy. The hyperparameter tuning effect of MBAS on DT, RF, and KNN is shown in Figure 11. It can be clearly seen that with the increase in the number of iterations, the changing trend of the three machine learning models decreases rapidly and then becomes stable. Among these, the RMSE value of the RF model has the best convergence effect, which proves that the hyperparameter tuning effect of MBAS on RF is better than DT and KNN.

**Figure 12:**
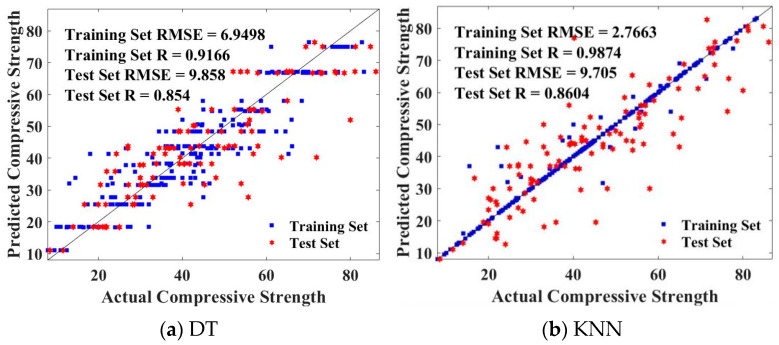
Fitting effect between predicted values and actual values of DT and KNN.

Figure 12 shows the fitting effect of DT-MBAS and KNN-MBAS on the predicted and actual values of the CS of geopolymer concrete. Obviously, DT-MBAS has poor consistency between the predicted and actual values, and the RMSE values (6.9498 and 9.858) of the training set and the test set are both high, and the R values (0.9166 and 0.854) are both low. Although the training set of KNN-MBAS has a low RMSE value (2.7663) and a high R value (0.9874), due to the overfitting phenomenon, the prediction effect of the test set is poor, with a RMSE value of 9.705 and R value of 0.86045. On the whole, RF-MBAS has higher accuracy in predicting the CS of geopolymer concrete than DT-MBAS and KNN-MBAS.

**Figure 13:**
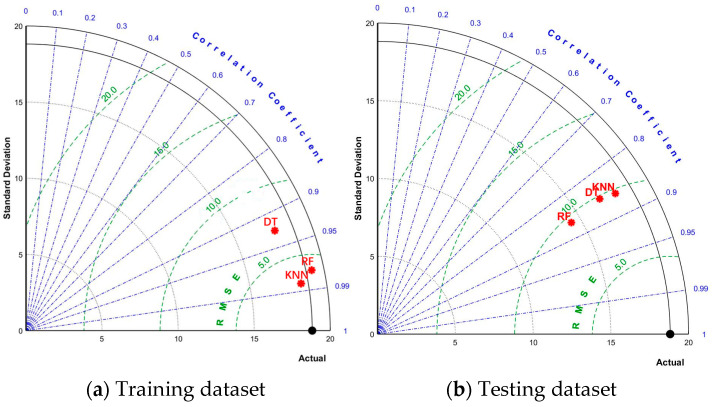
Comparison of RSME values and R values of three models.

Figure 13 more intuitively shows the comparison of RMSE and R values for the training dataset and test dataset of the three machine learning models evaluating the CS of geopolymer concrete. In the training dataset, the KNN model performs the lowest RMSE and highest R among the three models. However, due to the overfitting phenomenon, the R value of the KNN model in the test dataset is low and the RMSE value is high, and the RF model has the highest R and the lowest RMSE in the test dataset, proving that RF is the most accurate model to predict the CS of geopolymer concrete among the three models.

In order to further verify the accuracy of RF-MBAS for the prediction accuracy of the CS of geopolymer concrete, this study compared the mean squared error (MSE), RMSE, mean absolute error (MAE), mean absolute percentage error (MAPE), R, median prediction error (MPE), and performance index (PI) of three machine learning models, and the results are shown in Table 3. The MSE, RMSE, MAE, and MAPE values of RF-MBAS are 93.382, 9.663, 6.999, and 0.162, respectively, which all rank first among the three models, and R is 0.867, ranking first among the three models; only the MPE is higher than that of KNN-MBAS, ranking second, but the total score of all indexes of RF-MBAS is 8, the lowest among the three models, which proves again that RF-MBAS has the highest prediction accuracy for the CS of geopolymer concrete among the three models.

### 3.4. Importance Analysis and Sensitivity Analysis of Input Variables

**Figure 14:**
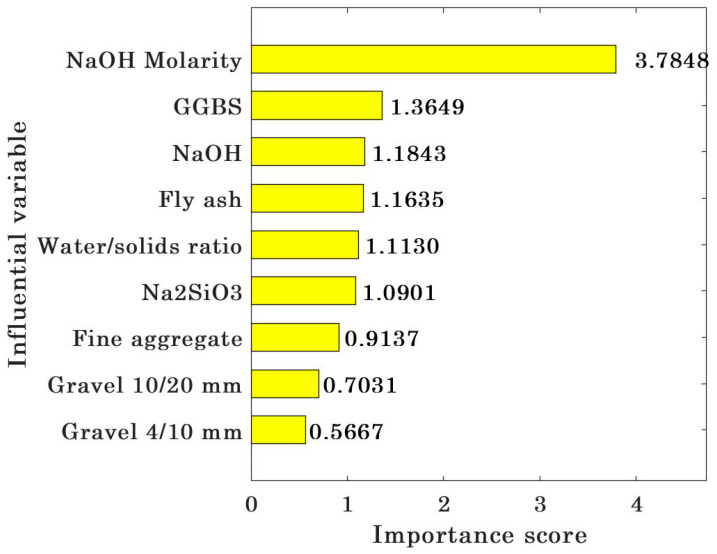
Importance analysis of input variables.

**Figure 15:**
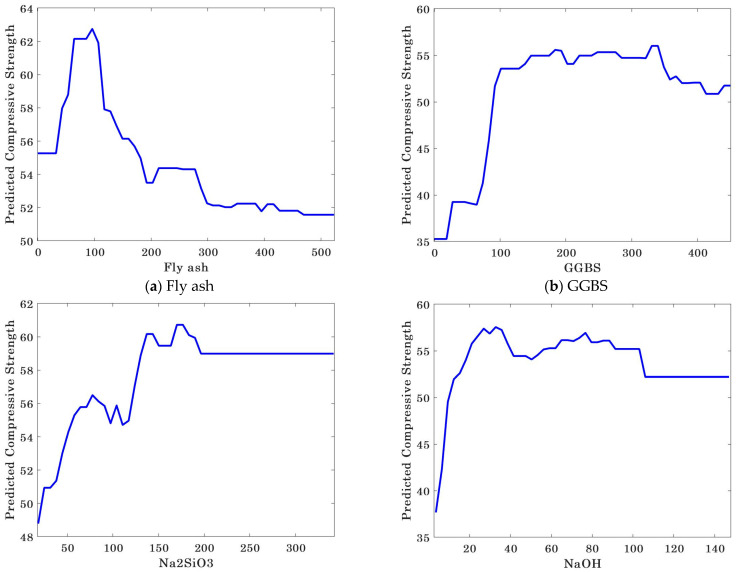

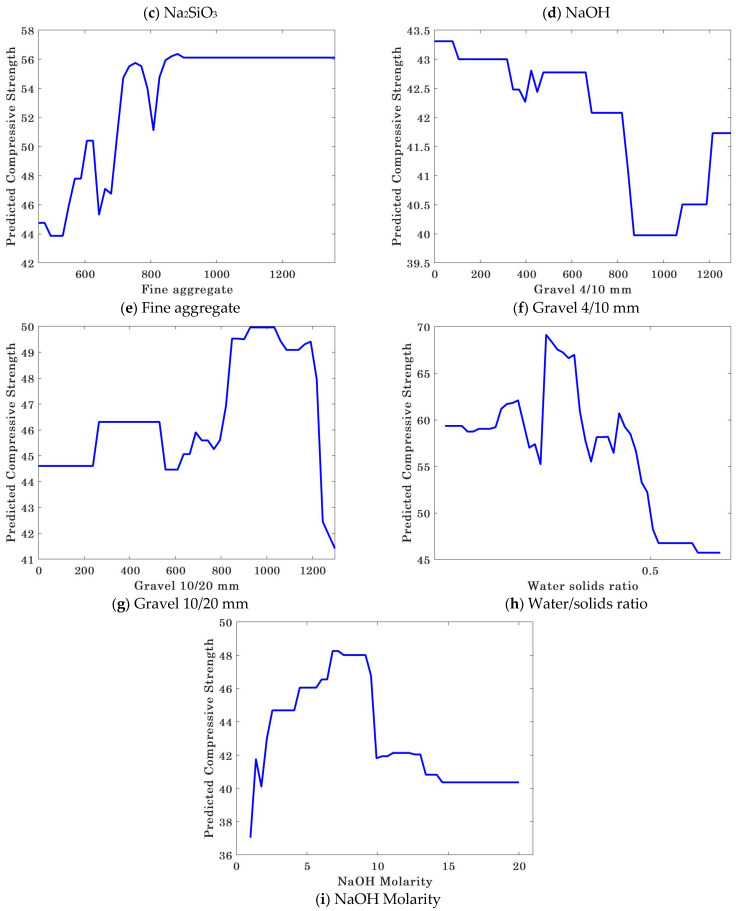
Sensitivity analysis of input variables.

The analysis result of the importance of input variables to the CS of geopolymer concrete is shown in Figure 14. As can be seen from the figure, the importance score of input variables to the CS decreases in the order of NaOH molarity (3.7848), GGBS (1.3649), NaOH (1.1843), fly ash (1.1635), water/solids ratio (1.1130), Na_2_SiO_3_ (1.0901), fine aggregate (0.9137), grade 10/20 mm (0.7031), and grade 4/10 mm (0.5667). Figure 15 shows the sensitivity analysis of the input variables of the CS of geopolymer concrete. It is obvious that all input variables have a high sensitivity to the CS of geopolymer concrete, and NaOH molarity, GGBS, and NaOH have a strong impact on the CS of geopolymer concrete, while grade 10/20 mm and grade 4/10 mm have a weak sensitivity. Hence, to prepare geopolymer concrete with high CS, engineers should pay more attention to the active substances and alkaline activators [29,93].

### 3.5. Graphical User Interface (GUI) Development

**Figure 16:**
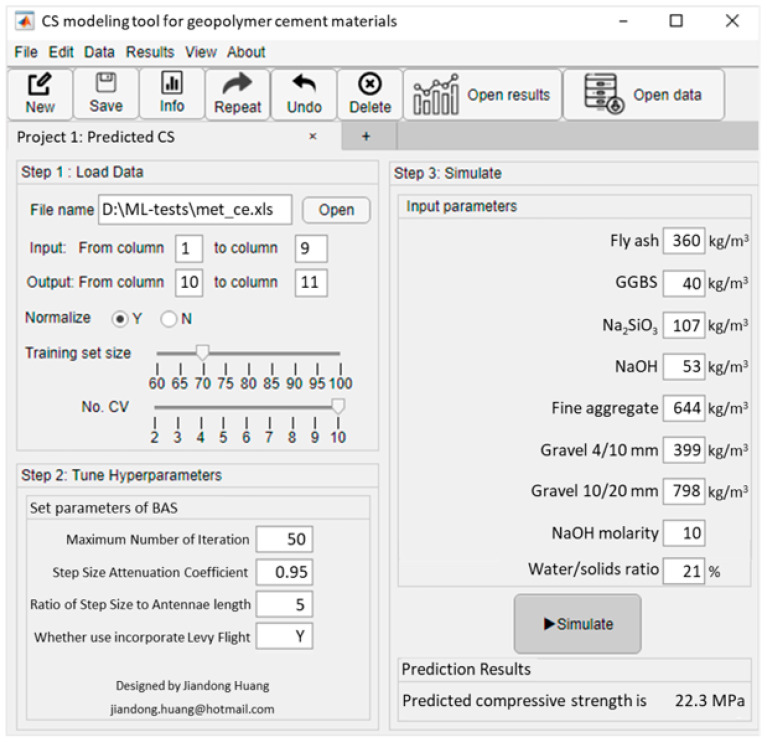
Screenshots of developing GUI.

**Figure 17:**
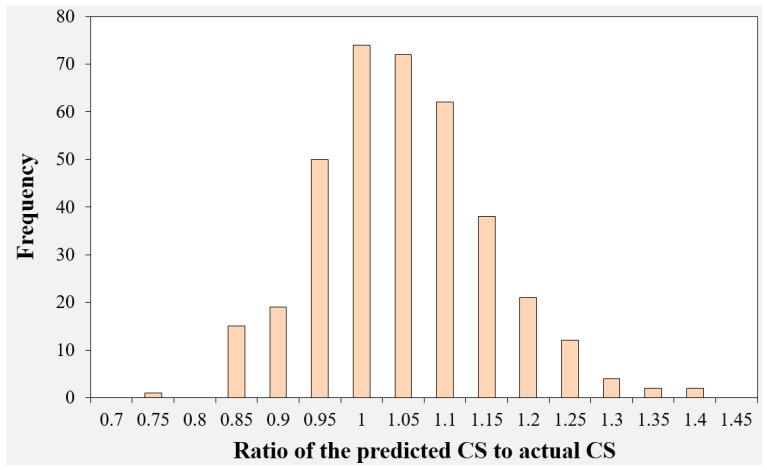
Histogram plot of the ratio of predicted compress strength to actual CS of geopolymer concrete.

In this study, a graphical user interface (GUI) for evaluating the CS of geopolymer concrete is designed based on MATLAB, which provides an outdoors-friendly tool for civil engineers. As shown in Figure 16, the user can complete hyperparameter tuning, model training, and validation in the GUI, and finally use it to determine the CS of geopolymer concrete based on the input parameters. To validate the GUI, the author entered fly ash, GGBS, Na2SiO3, NaOH, fine aggregate, gravel 4/10 mm, gravel 10/20 mm, NaOH molarity, and the water/solids ratio as 360kg/m^3^, 40kg/m^3^, 107kg/m^3^, 53 kg/m^3^, 644 kg/m^3^, 399 kg/m^3^, 798 kg/m^3^, 10, and 21%, and the predicted CS of the gels is 22.3MPa. To further verify the accuracy of the prediction of GUI, the author tested all data of the test set using GUI, and Figure 17 shows the histogram of the ratio of the predicted and actual values. It can be seen from the figure that the ratio between the predicted value and the actual value is 1–1.05, and the ratio between the predicted value and the actual value of most samples is 0.9–1.15, and only a few samples are 0.7–0.75 and 1.35–1.5. As a whole, the evaluation accuracy of GUI for the CS of geopolymer concrete was high.

## 4. Conclusions and Discussion

The intent of this study is to design an intelligent optimization tool for evaluating the CS of geopolymer concrete. The popularization and application of geopolymer concrete in the construction industry is conducive to promoting the development of eco-friendly building materials. This study has a positive impact on promoting the application of geopolymer concrete. To gain a high-performance model, an effective technique was developed using the RF and MBAS models for the prediction of the CS of geopolymer concrete. The hyperparameters were tuned using the MBAS, and improved by Levy flight and the adaptive weight method in order to overcome the shortage of BAS. The results of the RF-MBAS were also compared to those of DT-MBAS and KNN-MBAS to evaluate the dependability of RF-MBAS. The main results are as follows:

(1) To avoid the limitation that traditional BAS is prone to fall into local optimum due to premature convergence, Levy flight and the adaptive weight method were used to improve BAS. The rate of convergence and the significant reduction in RMSE values prove the efficiency and accuracy of MBAS;
(2) In terms of the prediction of the CS of geopolymer concrete, there is a good agreement between the predicted values and the actual values, and the error is small, which proves the feasibility of RF-MBAS in this study, and the comparison with DT-MBAS and RF-MBAS also confirms this view;
(3) NaOH molarity and GGBS are the variables that have the greatest impact on the CS of geopolymer concrete, based on the dataset designed in the present study. NaOH and fly ash also show certain influence ability, but the change in the size of gravel (gravel 10/20 mm and gravel 20/40 mm) has a weak influence on the CS;
(4) The high density of samples with a ratio close to 1 between the predicted and the actual values proves that the GUI developed using RF-MBAS is reliable;
(5) By developing an accurate and convenient tool for the CS of geopolymer concrete, the research will promote the application of economical and environmentally friendly geopolymer concrete and promote the construction industry movement towards sustainable development.

In this study, a hybrid MBAS and RF machine learning model is proposed to develop an intelligent system for evaluating the CS of geopolymer concrete, and the accuracy of the model is verified. In future research, it is necessary to develop multi-objective optimization models to simultaneously optimize the mechanical properties, economic properties, carbon emissions, and other competing objectives of geopolymer concrete.

## Figures and Tables

**Table 1:** Structural classification of gels.

No.	Name	Type	Aggregation Unit Structure
1	Poly(sialate)	PS	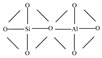
2	Poly(sialate-siloxo)	PSS	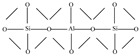
3	Poly(sialate-disiloxo)	PSDS	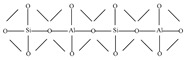

**Table 2:** Statistical analysis of input variables dataset.

Data	Statistical Indicators	Inputs									Output CS
		Fly Ash	GGBS	Na_2_SiO_3_	NaOH	Fine Aggregate	Gravel 4/10 mm	Gravel 10/20 mm	W/S	NaOH Molarity	
Training dataset	Min	0	0	18	3.5	460	0	0	0.12	1	8
	Max	523	450	342	147	1360	1293.4	1298	0.63	16	86.06
	Range	523	450	342	143.5	900	1293.4	1298	0.51	15	78.06
	Median	120	280	108	51.14	742	208	789	0.34	8.4	42
	Average	176.95	222.64	113.19	55.05	735.68	286.08	736.97	0.35	8.15	43.19
	St. Dev	168.50	163.05	47.54	31.56	136.37	370.19	356.92	0.11	4.4	18.15
Test dataset	Min	0	0	18	4.3	459	0	0	0.2	1	9.5
	Max	523	450	293	143.33	990	1209	1280	0.53	20	78.5
	Range	523	450	275	139.03	531	1209	1280	0.33	19	69
	Median	120	277.50	104.41	49.74	704.53	210.16	802	0.34	10	43
	Average	173.65	225.41	110.38	50.63	711.26	302.80	736.68	0.34	8.47	44.8
	St. Dev	164.09	156.90	47.14	32.45	105.75	387.21	371.97	0.10	4.43	16.32

**Table 3:** Analysis of the evaluation indexes of three models.

Models	Evaluation Indexes							Total Rank
	MSE	RMSE	MAE	MAPE	R	MPE	PI	
RF-MBAS	93.38(1)	9.66(1)	7.00(1)	0.16(1)	0.87(1)	4.79(2)	0.160(1)	8
DT-MBAS	97.18(3)	9.86(3)	7.52(3)	0.18(2)	0.85(3)	6.19(3)	0.165(3)	20
KNN-MBAS	94.19(2)	9.71(2)	7.09(2)	0.18(2)	0.86(2)	4.68(1)	0.162(2)	13

## Data Availability

The data presented in this study are available on request from the corresponding author.
